# Diet and Sleep Physiology: Public Health and Clinical Implications

**DOI:** 10.3389/fneur.2017.00393

**Published:** 2017-08-11

**Authors:** Sarah Frank, Kelli Gonzalez, Lorraine Lee-Ang, Marielle C. Young, Martha Tamez, Josiemer Mattei

**Affiliations:** ^1^Department of Global Health and Population, Harvard T.H. Chan School of Public Health, Boston, MA, United States; ^2^Department of Environmental Health, Harvard T.H. Chan School of Public Health, Boston, MA, United States; ^3^Department of Social and Behavioral Sciences, Harvard T.H Chan School of Public Health, Boston, MA, United States; ^4^Department of Medical Education, Icahn School of Medicine at Mount Sinai, New York, NY, United States; ^5^Department of Nutrition, Harvard T.H. Chan School of Public Health, Boston, MA, United States

**Keywords:** diet and sleep, nutrition and sleep, sleep quantity, sleep quality, sleep physiology

## Abstract

This mini-review examines the complex relationship between diet and sleep and explores the clinical and public health implications of the current evidence. Dietary quality and intake of specific nutrients can impact regulatory hormonal pathways to alter sleep quantity and quality. Sleep, in turn, affects the intake of total energy, as well as of specific foods and nutrients, through biological and behavioral mechanisms. Initial research in this field focused primarily on the effects of short sleep duration on nutritional quality. However, more recent studies have explored the dynamic relationship between long sleep duration and diet. Current evidence suggests that extremes of sleep duration alter sleep patterns, hormonal levels, and circadian rhythms, which contribute to weight-related outcomes and obesity, and other risk factors for the development of chronic disease such as type 2 diabetes and cardiovascular disease. These patterns may begin as early as childhood and have impacts throughout the life course. Given that non-communicable diseases are among the leading causes of death globally, deeper understanding of the interactions between sleep and nutrition has implications for both public health and clinical practice.

## Introduction

**Figure 1:**
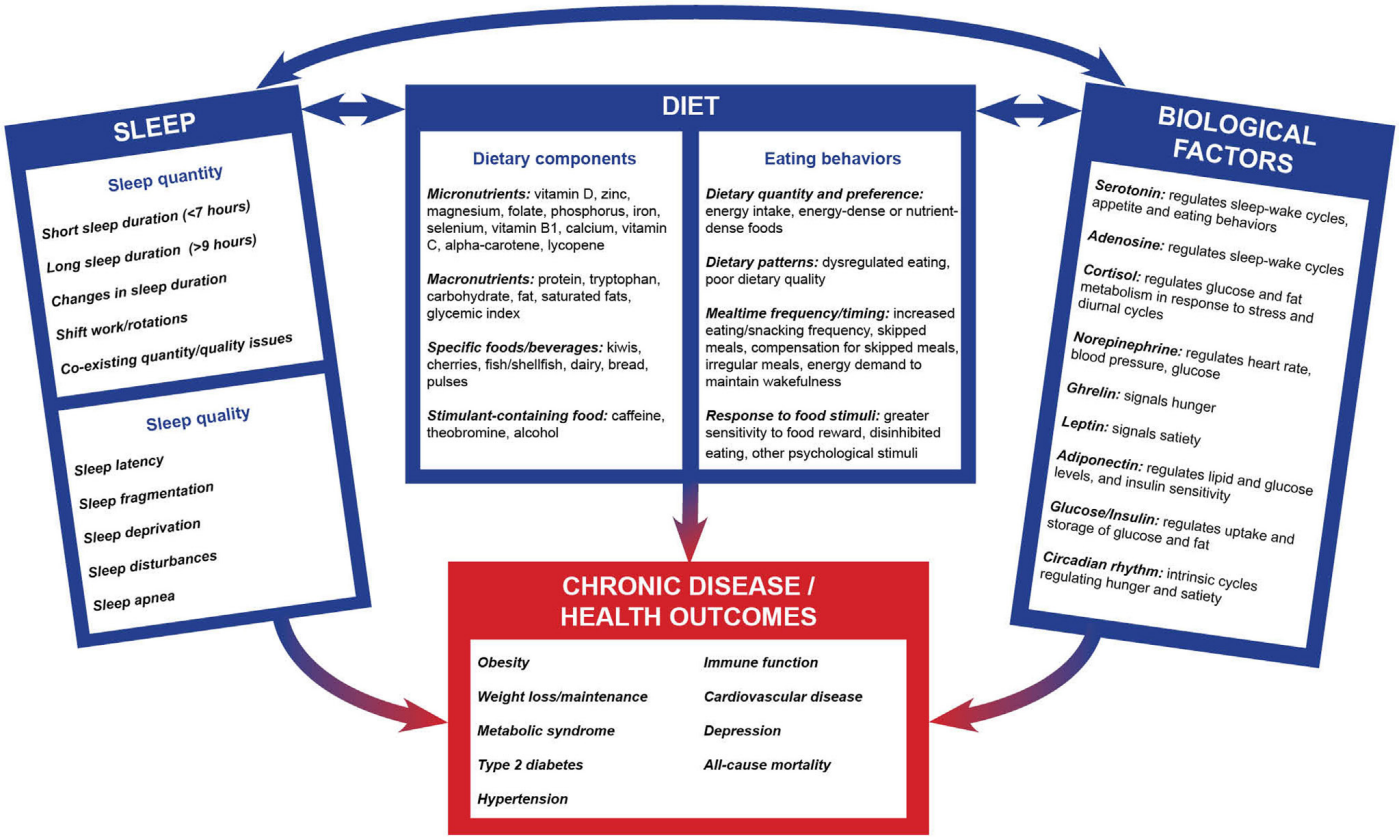
Conceptual framework for the interconnections between dietary factors, sleep, and disease. The complex, bidirectional, relationship between sleep components, dietary composition, behavioral factors, and biological factors are theorized to affect the development of chronic diseases and other health outcomes. Intake of nutrients and foods and dietary behaviors are correlated with components of sleep quality and quantity. Dietary components and eating behaviors are regulated by biological factors, which in turn may impact nutritional status. Similarly, sleep quantity and quality result in biological factors being modulated, and these biological factors control sleep factors in return. Dietary components, sleep components, and biological factors have subsequent independent as well as interactive effects on downstream health outcomes. Notably, this multifaceted interaction is evident early in life and continues throughout the life course.

In contrast to other lifestyle risk factors of chronic disease, sleep has not been accorded the same amount of attention in public health or clinical research and practice until recently. There exist complex processes linking sleep duration, quality, and behaviors to both nutrition and risk of chronic disease (Figure 1). Currently, the evidence suggests a bidirectional relationship between sleep quality and duration and diet. These sleep components and their interactions with diet subsequently affect the risk of developing chronic disease. Here, we summarize the main evidence for these complex relationships. We also review the evidence for the effects of diet quality, sleep-promoting foods, and dietary composition on sleep outcomes. We then consider the influence of sleep quality and quantity on risk of chronic disease, such as obesity and weight-related outcomes, type 2 diabetes, and cardiovascular disease (CVD). We extend this discussion by examining the relationships between sleep and diet throughout the life course. Finally, we discuss the clinical and public health implications of this evidence and suggest possible directions for future research. Of note, this mini-review summarizes the literature on human studies, while recognizing that there are data from animal experimental studies that have explored potential mechanisms relating diet and sleep.

## Scientific Evidence

### Diet and Sleep

Dietary composition, with a focus on specific dietary components, has been shown to influence sleep duration, quality, and behaviors (1–4). We cite studies analyzing the role of macronutrients, micronutrients, and whole foods on sleep (Table 1); most have been small trials or cross-sectional studies conducted in healthy adults. For macronutrients, low fiber, high-saturated fat, and sugar intake were associated with lighter, less restorative sleep, over *ad libitum* diet, in an inpatient sleep crossover study (5). In another study, a high-carbohydrate/low-fat diet was associated with poorer sleep quality vs. a normal balanced diet or a low-carbohydrate/high-fat diet (6). Protein and carbohydrate deficiencies have also been associated with shorter sleep duration (4). A study found that consuming high-glycemic index carbohydrate meals approximately 4 h before bedtime decreased sleep latency, or the time to sleep initiation, which was attributed to an increase in tryptophan after carbohydrate consumption (7). Tryptophan is an amino acid precursor to the sleep-regulating hormone serotonin and is often mentioned as important in the proposed relationships between diet and sleep (8). Furthermore, another study found that evening dietary increases in tryptophan intake improved sleep in adults with sleep disturbances and enhanced alertness in the morning, most likely as a result of improved sleep quality (9, 10).

**Table 1:** Example of studies linking dietary components and sleep outcomes.

Study design	Participants	Dietary component	Sleep outcome	Reference
**Macronutrients or energy intake**				
Laboratory-based intervention	Young healthy males	High-carbohydrate/low-fat (vs. low-carbohydrate/high-fat or balanced isocaloric diets)	Less slow-wave sleep	Phillips et al. (6)

Laboratory-based experiment	Young healthy males	High-glycemic index carbohydrate meals (vs. low glycemic index)	Shortening of sleep onset latency	Afaghi et al. (7)

Cross-sectional survey	Adults (general population)	Fat intake	Decreased sleep duration	Shi et al. (41)

Parallel, randomized, controlled, open label trial	Men with obesity and moderate to severe obstructive sleep apnea	Liquid very low energy diet followed by gradual normal food (vs. usual diet)	Improved obstructive sleep apnea	Johansson et al. (43)

Longitudinal cohort	3-month-old infants followed-up until age 2	Higher energy intakes at the evening meal	Longer sleep duration	Diethelm et al. (63)
		Carbohydrates (especially from high GI or high GL foods)		

Randomized, crossover intervention	Inpatient normal weight adults	Low fiber, high-saturated fat, and sugar intake vs. *ad libitum*	Lighter, less restorative sleep with more arousals	St-Onge et al. (1, 5)

**Micronutrients**				
Double-blind, placebo-controlled trial	Healthy young adults	Tryptophan-rich diet (vs. tryptophan-low placebo protein)	Reduced sleepiness and sustained alertness in the following morning	Markus et al. (9)

Double-blind, placebo-controlled clinical trial	Older adults with primary insomnia in a long-term care facility	Food supplement with melatonin, magnesium, and zinc (vs. placebo)	Improved sleep quality (getting to sleep, quality of sleep, alertness upon awakening) and sleep time	Rondanelli et al. (12)

Cross-sectional analysis	Adults (general population)	Deficiency in vitamin B1, folate, iron, zinc, phosphorus, magnesium, and selenium	Shorter sleep duration	Grandner et al. (4)

Cross-sectional analysis	Adults (general population)	Deficiency in alpha-carotene, selenium, calcium, vitamin D, lycopene, and vitamin C	Difficulty falling asleep, sleep maintenance, or non-restorative sleep	Grandner et al. (11)

Double-blind, clinical trial	Adults with sleep disorders	Vitamin D supplement (vs. placebo)	Improved sleep quality, reduced sleep latency, increased sleep duration, and improved subjective sleep quality	Majid et al. (13)

Randomized, double-blinded, placebo-controlled parallel group trial	Healthy adults	Zinc-rich food (vs. zinc-, and astaxanthin-rich food or placebo supplemented with zinc-enriched yeast and astaxanthin oil, or placebo)	Decreased time needed to fall asleep and improved sleep efficiency	Saito et al. (14)

**Foods**				
Randomized, double-blind, placebo-controlled, crossover	Healthy elderly	Fermented milk drink (vs. placebo drink)	Improved sleep efficiency and number of wakening episodes	Yamamura et al. (21)

Randomized, double-blind, crossover	Healthy older adults with chronic insomnia	Tart cherry juice beverage (vs. placebo)	Reduction in insomnia severity	Pigeon et al. (24)

Laboratory-based experiment	Middle-aged and elderly healthy adults	Jerte Valley cherry cultivars	Beneficial effects on sleep time, total nocturnal activity, assumed sleep, and immobility	Garrido et al. (25)

Free-living, self-controlled diet	Adults with self-reported sleep disturbance	Kiwi fruit	Improved sleep onset, duration, and efficiency	Lin et al. (26)

Randomized, placebo-controlled	Inpatient male adults	Atlantic salmon (vs. alternative meal, i.e., pork, beef, and chicken)	Better daily functioning	Hansen et al. (22)

Cross-sectional analysis	Middle-aged and older adults	Oily fish consumption	Better sleep quality	Del Brutto et al. (23)

**Eating behaviors**				
Cross-sectional survey	Middle-school children	“Unhealthy eating habits and environments” and “snacking between meals and after supper” (identified by factor analysis)	Shorter sleep and poor sleep quality	Khan et al. (65)

**Mixed dietary components**				
Cross-sectional analysis	Healthy adult men	Percentage of energy from protein, energy-adjusted intake of sodium, vitamin D, and vitamin B12, intake of bread, pulses, and fish and shellfish	Longer sleep duration	Komada et al. (27)

Micronutrients intake have also been suggested to affect sleep patterns. For example, associations have been reported for deficiencies in vitamin B1, folate, phosphorus, magnesium, iron, zinc, and selenium with shorter sleep duration (4), lack of alpha-carotene, selenium, and calcium with difficulty falling asleep, low intake of vitamin D and lycopene with sleep maintenance, and low intake of calcium and vitamin C with non-restorative sleep (11). Short-term trials have shown that nightly intake of melatonin, magnesium, or zinc improved sleep quality in long-term care facility residents with insomnia (12), and use of vitamin D supplement resulted in better outcomes for sleep quality, sleep latency, and sleep duration in adults with a sleep disorder (13). The evidence for zinc is further supported by another randomized, double-blinded, placebo-controlled trial in healthy adults that showed that zinc-rich foods improved sleep onset latency and sleep efficiency over placebo (14). Moreover, optimal magnesium levels, rather than levels above or below the clinically recommended range, have been found in a mice study to be required for normal sleep regulation. The exact mechanisms through which these micronutrients may affect sleep remains unclear (15). Micronutrients have also been proposed as mediators of diet–disease associations; for example, carotenoids and vitamin D mediate associations between sleep duration and waist circumference or systolic blood pressure, and vitamin C mediates the sleep duration–diastolic blood pressure relationship (16).

Intake of stimulant-containing foods and beverages similarly affects elements of sleep. Caffeine and theobromine are competitive antagonists to adenosine, a hormone that regulates sleep–wake cycles (17). While caffeine and theobromine provide immediate energy after consumption, there are also longer lasting effects consequences that alter sleep patterns for many hours after intake, including prolonged sleep latency, reduced total sleep time, sleep inefficiency, worsened perceived sleep quality, and REM sleep behavior disorder (18, 19). In addition, alcohol, which is often regarded as a sedative, has a nuanced impact on sleep. Consumption of alcohol decreases sleep latency and may disrupt sleep later due to its ability to influence levels of serotonin and norepinephrine (20).

There is evidence that particular whole foods affect sleep. For example, milk, fatty fish, cherries, and kiwis have been associated with beneficial effects on sleep outcomes (1, 21–26). The relatively high content of tryptophan found in some of these specific foods may be responsible for these observed associations (7, 8). Intake of bread, pulses, and fish and shellfish has been positively correlated with sleep duration in men (27). Finally, there is evidence to suggest that changes in daily dietary composition and eating behaviors can subsequently affect elements of sleep (28).

### Sleep and Chronic Disease

Growing evidence suggests that sleep patterns, such as short (<7 h) and long (>9 h) sleep duration, can impact risk of chronic disease (29). Short sleep has received more scrutiny, and it has been associated with an increased risk of obesity, type 2 diabetes, and CVD (29); it also impairs glucose metabolism, which aggravates the risk of type 2 diabetes (30). Researchers hypothesize that short sleep duration may interfere with the body’s restorative processes that occur during sleep, leading to biological and behavioral risk factors for chronic disease development (31).

Biologically, sleep influences the circulating levels of the hunger signaling hormones ghrelin and leptin. Ghrelin indicates hunger and leptin signals satiety; sleep deprivation causes high levels of ghrelin and low levels of leptin. Therefore, the hormonal imbalance of ghrelin and leptin may induce overeating behaviors (30, 32). Furthermore, adiponectin, a secretory product of adipose tissue that is found in lower levels in the plasma of individuals with obesity, is inversely associated with sleep duration in teenage girls (33). Sleep deprivation also causes greater neuronal activation in response to food stimuli, which results in increased motivation to seek food with high energy intake, particularly energy-dense foods high in fat and sugar (1).

Another pathway by which sleep deprivation and disorders contribute to metabolic dysregulation is through activation of the hypothalamic–pituitary–adrenal (HPA) axis, which deregulates neuroendocrine parameters such as cortisol, leading to downstream increases in glucose and insulin and decreases in adiponectin levels (34). The HPA-axis pathway, along with increased sympathetic nervous system activity and inflammatory responses, has been implicated in the relationship between short sleep duration and increased risk for hypertension, coronary heart disease, recurrent acute coronary syndrome, and heart failure (35).

Circadian disturbances are another possible mechanism linking short sleep behaviors (such as shift work and sleep deprivation) and dietary behaviors and intake (meals irregularity and infrequency) to weight-related outcomes (36). Small laboratory studies conducted with healthy adults have established the presence of intrinsic circadian rhythms regulating hunger, satiety, and food-specific appetite (37, 38), as well as increases in energy expenditure with sleep deprivation or decreases with wakefulness and recovery events (39). Moreover, it has been reported that men have higher energy intake during sleep restriction and late-night hours, making them more susceptible to weight gain during sleep loss (40).

As for behavioral mechanisms, both short sleep duration and poor sleep quality are correlated with increased energy intake, poor diet quality, and dysregulated dietary patterns, which can lead to weight gain (2, 3, 41). Other proposed behaviors include more time and opportunities for eating, psychological distress, greater sensitivity to food reward, disinhibited eating, more energy needed to sustain extended wakefulness, and changes in appetite hormones (2). Indeed, those who experience short sleep duration demonstrate more irregular eating patterns, including more frequent, smaller, and energy-dense foods during non-regular mealtimes (3).

Sleep deficiency may thus promote excess energy intake by affecting both eating behaviors and dietary composition (2). There is an established association between short sleep duration and higher total energy and fat intake (3). For example, individuals sleeping less than 7 h per night have a significantly higher proportion of total energy intake from fat, compared to those sleeping the recommended 7–9 h per night. In fact, the evidence suggests an inverse dose–response relationship between sleep and fat intake (41).

While the association between sleep and weight gain has been well studied and appears robust (42), the effect on weight loss remains unclear. Randomized clinical trials have suggested that weight loss and weight maintenance through diet and lifestyle interventions can contribute to sleep improvements (43–47), although these have been mostly conducted in patients with preexisting sleep disorders. One clinical trial reported a direct association between sleep duration and successful weight loss (48). However, further research is needed to elucidate the directionality of these associations, and whether sleep improvement could lead to weight loss in the general population.

Finally, there is emerging evidence regarding the association between long sleep duration, usually defined as more than 9 h of sleep, and chronic disease (49–51). Long sleep duration has been associated with higher risk of CVD, type 2 diabetes, depression, obesity, and chronic kidney disease in trials and observational studies (29, 49–53). However, the mechanisms for these relationships are not clear (49, 50). There may be unobservable reverse causation bias, as these conditions may cause sleep disruptions, such as sleep apnea and sleep fragmentation, that are then associated with long sleep duration. Coexisting poor sleep quality and longer sleep duration have also been shown to be associated with higher cortisol reactivity in adolescents in a cross-sectional study (54) and higher incident coronary heart disease in a cohort of women (55). Moreover, evidence from prospective studies suggests that changes in sleep duration may increase the risk of metabolic syndrome, type 2 diabetes, and CVD mortality as well as of all-cause mortality (56–58). Studies have shown that workers with habitual changes in their shift rotations, and thus in their sleep patterns, have physical inactivity, overweight, sleep deprivation, increased cortisol secretion, and higher inflammation (59–61). Therefore, current evidence suggests that the relationship between sleep duration and risk of chronic disease is J-shaped, such that “extremes of sleep duration” may better predict elevated chronic disease risk (62).

### Diet and Sleep throughout the Life Course

The complex relationship between diet, sleep, and risk factors for chronic disease becomes evident early in life and continues throughout the life course. The impact of dietary composition on sleep patterns has been observed in early childhood. In a cohort of 1- and 2-year-old children, higher energy intake during the evening meal was associated with longer sleep duration (63). Studies of sleep patterns in children also highlight the impact on biological risk factors—particularly the dysregulation of ghrelin and leptin—that contribute to chronic disease risk. A longitudinal study that evaluated sleep duration and leptin found that chronic short sleep duration was associated with lower levels of leptin later in childhood and was exacerbated in girls with greater body adiposity (64). There is also evidence that eating behaviors affect sleep in children. A cross-sectional study found that children who snack in-between meals or after dinner demonstrate decreased sleep duration and quality (65). Furthermore, decreased sleep duration in children has been associated with higher obesity risk (66).

Similar to the pattern observed in young children, adolescents that report short sleep duration have elevated ghrelin and relatively low leptin levels (32). Adolescents are at particularly high risk for short sleep duration and currently report the highest prevalence of insufficient sleep (68.9%) (67). There are several unique behavioral risk factors that may account for this finding, including increased electronic device use and unhealthy diets (68–72). Nearly one-quarter of adolescents report using an electronic device “constantly,” and 72% report bringing their cellphones into their bedrooms and using them while trying to fall asleep (68). Increased screen time has been associated with poorer sleep quality, unhealthy eating behaviors, and decreased physical activity (69, 70). In addition, adolescents with short sleep duration eat significantly fewer servings of fruits and vegetables and have increased odds of fast food consumption, relative to adolescents who sleep for 8 h per night (71). Similarly, adolescents sleeping fewer than 8 h per night consumed a higher proportion of energy from fat and a lower proportion of energy from carbohydrates compared with adolescents sleeping more than 8 h (72). Compounding these risk factors, adolescents are frequently the target audience for marketing of energy-dense, nutrient-poor food, and beverage products (73). The combination of short sleep duration and an obesogenic environment among adolescents may contribute to developing eating behaviors and dietary choices that increase the risk of further sleep disturbances and chronic disease. In fact, sleep deprivation in adolescents has subsequently been associated with a higher risk for obesity, decreased insulin sensitivity, and hypertension (74, 75).

Much of the research regarding the relationship between diet and sleep has been conducted in healthy, young or middle-aged adult populations. There is less evidence regarding the relationship between sleep and nutrition in the elderly, or in subgroups with preexisting conditions. The limited research conducted with the elderly echoes that found in younger populations. Among the elderly, poor sleep quality has been associated with obesity, hypertension, metabolic syndrome, and type 2 diabetes (76–78). However, many of these studies are cross-sectional, and issues of reverse causality are especially relevant in elderly populations, who are already at elevated risk of developing these conditions.

## Implications for Public Health and Clinical Practice

According to the National Sleep Foundation, both diet quality and sleep duration are poor and have been declining steadily in the U.S. population (66, 79). In 2010, 42% of U.S. adults and more than 50% of U.S. children reported insufficient sleep (80). Concurrently, lifestyle risk factors that may be influenced by sleep and are protective against chronic disease have been on the decline: the percent of the U.S. population adhering to five healthy lifestyle habits decreased from 15% in 1988 to 8% in 2006 (81). The changes in the U.S. reflect global patterns of reported sleep disturbances and shifts toward unhealthier lifestyle behaviors, especially in low-income Asian and African countries (82). These trends highlight the importance of translating the existing scientific evidence focusing on the relationship between sleep patterns and nutrition into messages, programs, and interventions that the public can easily understand and utilize to prevent chronic disease.

Nevertheless, there are few official sleep recommendations available to guide health practitioners and the general population. In the U.S., the National Sleep Foundation has published age-specific, evidence-based recommendations for sleep duration to lower the risk of chronic disease (83). The American Academy of Sleep Medicine has also provided pediatric and adult recommendations for sleep duration (84, 85). The 2015 Dietary Guidelines for Americans include recommendations about physical activity and other aspects of a healthy lifestyle; however, they do not include recommendations on the integral relationship between diet and sleep (86). Considering the mounting literature, information on sleep should be incorporated into future iterations of the Dietary Guidelines for Americans to further enhance healthy lifestyle recommendations. Similarly, global agencies and other countries could dedicate more resources to this topic to provide population-wide recommendations on sleep and nutrition.

In addition, there should be efforts to incorporate sleep-related content into existing interdisciplinary programs that target nutrition and other relevant aspects of health. Initial work in this area has been promising. For example, in a community-based intervention focused on wellness, participants experienced improvements in dietary quality, sleep duration, and indicators of obesity (87). An intervention to prevent adverse sleep behaviors among pregnant women demonstrated a protective effect against obesity in the child’s first 2 years of life (81). Most encouraging, these improvements have been observed in individuals from a variety of cultural and socioeconomic backgrounds (87–89).

Additional strategies could help improve the approach to sleep and nutrition in the clinical setting. We recommend the following actions: (1) train and educate health-care professionals on the relationship between diet and sleep, particularly those caring for at-risk groups; (2) develop and apply rapid, validated screeners to assess diet composition, eating behaviors, and sleep patterns, to help identify and counsel at-risk patients; and (3) develop new and integrative therapies that account for the critical associations between diet and sleep.

### Future Research

Despite the evidence and public health recommendations presented here, there is still a gap in understanding the complex relationship between sleep, diet, and nutrition, and risk for chronic disease. Much of the evidence to date has been done in cross-sectional studies or small trials, making it difficult to define causal pathways between various dietary components and sleep or to generalize results. More research is needed to understand the mechanisms by which specific nutrients, foods, and eating behaviors impact quality and quantity of sleep. This could be achieved through laboratory studies, larger randomized clinical trials, and longitudinal analyses of diet and sleep outcomes. Similarly, these studies can help identify potential mechanisms that mediate the relationship between sleep, diet, and risk for chronic disease. Future research should seek to strengthen the evidence linking short and long sleep duration and risk of chronic disease. Such research would inform clinical and public health recommendations regarding the specific dietary and sleep behaviors associated with the lowest risk of developing chronic conditions.

At the population level, emerging research has explored the social determinants of sleep in the U.S. A recent study found that poor sleep quality was directly related to indicators of socioeconomic status and race/ethnicity, with African-American and Hispanic/Latino populations reporting the poorest sleep quality (90). Addressing the many barriers to optimal sleep and nutrition in underserved and minority populations is crucial to improving health on a population level. Research is needed to identify feasible, culturally appropriate interventions that target sleep- and nutrition-related health gaps. Finally, given the emergence of sleep disturbances as a global epidemic (82), research in other countries, especially low-income countries, is needed. Such research could be used to guide future sleep recommendations about sleep practices and nutrition in high-risk populations across the globe.

## Author Contributions

SF, KG, LL-A, and MY conceptualized the topic, researched and analyzed the background literature, and wrote the manuscript, including interpretations and conclusions. JM analyzed the content to prepare the table and portions of the figure and manuscript. MT and JM provided substantial scholarly guidance on the conception of the topic, manuscript draft and interpretation, and revised the manuscript critically for intellectual content. All the authors approved the final version of the manuscript, ensured the accuracy and integrity of the work, and agreed to be accountable for all aspects of the work.

## Conflict of Interest Statement

The authors declare that the research was conducted in the absence of any commercial or financial relationships that could be construed as a potential conflict of interest.
